# Exploratory study of PD-1 inhibitors with or without ruxolitinib for the treatment of adult EBV-associated hemophagocytic lymphohistiocytosis: a real-world data-based preliminary investigation

**DOI:** 10.1007/s10238-026-02136-0

**Published:** 2026-04-02

**Authors:** Zhao Chen, Wanting Li, Can Liu, Jianjun Chen, Xi Tan, Wei Li, Cheng Zhou, Fang Li, Ping Lei, Ming Zhou

**Affiliations:** 1 Department of Hematology, Hunan Provincial People’s Hospital, The First Affiliated Hospital of Hunan Normal University, Changsha, 410002 China; 2 Hunan Normal University , Changsha, 410081 China; 3 Laboratory of Hematology, Hunan Provincial People’s Hospital,The First Affiliated Hospital of Hunan Normal University, Changsha, 410002 China

**Keywords:** PD-1 Inhibitors, Ruxolitinib, EBV-associated hemophagocytic lymphohistiocytosis, Cytokine storm, Real-world study

## Abstract

To preliminarily analyze the clinical efficacy and safety of PD-1 inhibitors as monotherapy or in combination with ruxolitinib (RUX) for adult Epstein-Barr virus-associated hemophagocytic lymphohistiocytosis (EBV-HLH) based on real-world retrospective data. A retrospective case series analysis was performed on clinical data of 4 adult EBV-HLH patients treated with sintilimab (a PD-1 inhibitor) alone or combined with RUX at the Department of Hematology, Hunan Provincial People’s Hospital, from April 1, 2020 to February 1, 2024. Clinical efficacy, survival outcomes, dynamic changes of laboratory indicators and adverse reactions were observed. Among the 4 patients, 2 achieved complete remission (CR), 1 experienced treatment interruption due to non-adherence and was lost to follow-up, and 1 died of multi-organ failure induced by COVID-19, with a 1-year overall survival (OS) rate of 75.00%. All patients received sintilimab with a median treatment course of 9.5 cycles and a median infusion dose of 1900 mg; 3 patients were combined with RUX. After one cycle of treatment, the levels of alanine aminotransferase (ALT), aspartate transaminase (AST) and interleukin-10 (IL-10) decreased significantly, while white blood cell (WBC), hemoglobin (HGB) and platelet (PLT) levels increased significantly (all *P* < 0.05). Ferritin (Ferr) returned to the normal range and fibrinogen (Fbg) showed an upward trend. The number of peripheral blood T, B and natural killer (NK) cells increased in 3 patients who completed lymphocyte subset detection. No drug-related severe adverse reactions of grade 3 or above were observed in all patients. PD-1 inhibitors alone or combined with RUX show preliminary favorable efficacy and high tolerability in the treatment of adult EBV-HLH, which can effectively improve blood cell levels, control inflammatory responses and increase clinical remission rates. However, large-sample prospective studies are required to verify the long-term efficacy due to the small sample size of this study.

## Introduction

Hemophagocytic lymphohistiocytosis (HLH) is a rare syndrome characterized by excessive activation of T cells and macrophages, leading to a massive release of cytokines and causing a systemic hyperinflammatory response [1]. Epstein-Barr virus-associated hemophagocytic lymphohistiocytosis (EBV-HLH) is one of the most common subtypes, with clinical manifestations including persistent fever, cytopenias, hepatosplenomegaly, and elevated HLH biomarkers [2]. The HLH-94/04 regimen is currently a common treatment for adult EBV-HLH, but it has a short maintenance period, a high relapse rate, and can weaken the immune system’s ability to clear EBV. EBV-encoded BART11 and BART17-3p target and inhibit the transcriptional regulatory proteins FOXP1 and PBRM1, respectively. These proteins normally bind to the enhancer region of PD-L1 and suppress its expression. Therefore, by relieving FOXP1 and PBRM1’s transcriptional repression of PD-L1, BART11 and BART17-3p can upregulate PD-L1 expression and promote immune evasion in EBV-associated tumors [3]. Carbone A et al. [4]also found that in both EBV-associated and non-EBV-associated cases, viral infection can upregulate PD-L1.

The pathogenesis of EBV-HLH is closely linked to the impaired activity of cytotoxic T-cells (CTL) and NK cells that fail to clear EBV-infected cells, and EBV infection can activate the JAK-STAT signaling pathway in immune cells, leading to excessive secretion of proinflammatory cytokines such as IL-6, IL-10 and IFN-γ, which further amplifies the cytokine storm and aggravates the hyperinflammatory state of the body [5, 6]. Ruxolitinib, a selective JAK1/2 inhibitor, can block the JAK-STAT pathway to reduce various cytokines and control the hyperinflammatory response, thereby alleviating HLH [7], and has been confirmed to have good efficacy and safety in the treatment of pediatric and adult HLH [8–10]. At present, there are literature reports on the application of Ruxolitinib(RUX) in the treatment of hemophagocytic syndrome, including animal experiments, case reports, and clinical trials [11–13]. However, there is limited clinical research on the combined use of PD-1 inhibitors and RUX in treating EBV-HLH. This study conducts a preliminary exploratory analysis of clinical data from patients with EBV-HLH treated with PD-1 inhibitors alone or in combination with RUX, aiming to provide new insights into optimizing treatment strategies for this disease.

## Subjects and methods

### Study design

This is a single-center retrospective case series study. Clinical data were collected from adult patients with EBV-HLH who received off-label sintilimab alone or combined with ruxolitinib (RUX) in the Department of Hematology at Hunan Provincial People’s Hospital between April 1, 2020 and February 1, 2024. The study was approved by the Medical Ethics Committee of Hunan Provincial People’s Hospital (approval number: 2023 − 35.2), and written informed consent for the publication of clinical details was obtained from all patients or their legal representatives.

### Study subjects

Included patients were aged ≥ 18 years with primary or refractory/relapsed EBV-HLH (refractory: failure to achieve PR and above after 2 weeks of initial induction; relapsed: recurrence of HLH activity in a patient who has achieved PR and above after treatment), and the diagnosis of HLH had to meet at least 5 of the HLH-2004 diagnostic criteria [14, 15]. EBV-HLH diagnosis was confirmed by DNA polymerase chain reaction (PCR) with plasma EBV-DNA quantification ≥ 4.00 × 10² Copies/ml, or histopathological immunohistochemistry EBER positive, or magnetic bead sorting combined with qPCR to detect intracellular EBV-DNA quantification ≥ 500 Copies/1 million cells. All patients had a performance status (PS) score ≤ 4, no allergy to PD-1 inhibitors, Ruxolitinib (RUX), or any of their active ingredients or excipients, and the patients and their families were informed of the differences between the exploratory regimen and traditional HLH-94/04 regimen and signed informed consent.

Patients were excluded if they were pregnant or breastfeeding, had HLH clearly associated with malignant tumors based on tissue pathology, had other types of malignancies or congenital immune deficiencies.

### Treatment regimens

#### Conventional chemotherapy

Individualized conventional chemotherapy regimens were selected according to the patients’ examination results and specific conditions, including the DEP regimen (Doxorubicin 40 mg on day 1 + Etoposide 100 mg on day 1 + Methylprednisolone 112 mg on days 1–3 and 11.2 mg on days 4–14), the L-DEP regimen (Liposomal Doxorubicin 40 mg on day 1 + Etoposide 125 mg on day 1 + Methylprednisolone Sodium Succinate 780 mg on days 1–3, 39 mg on days 4–7, 13 mg on days 8–10, 5 mg on days 11–14 + Pegaspargase 1000 IU intramuscularly on day 3), the E-CHOP regimen (Etoposide 100 mg on days 2–3 + Cyclophosphamide 1200 mg on day 1 + Doxorubicin 40 mg on day 1 + Vincristine 40 mg on day 1 + Dexamethasone 15 mg on days 2–5), and the EP regimen (Etoposide 225 mg every 2 weeks + Dexamethasone Acetate 15.75 mg daily).

#### PD-1 inhibitors and ruxolitinib (RUX)

Sintilimab (manufactured by Innovent Biologics, specification: 100 mg/10 ml per vial) was used as the PD-1 inhibitor, with a dosage of 200 mg administered intravenously every 3 weeks for induction therapy. On the day of PD-1 inhibitor administration, corticosteroids were avoided. If fever occurred the day after administration, a temporary dose of 40 mg Methylprednisolone intravenously could be used for anti-inflammatory purposes. Treatment was discontinued if grade ≥ 3 immune-related adverse events occurred or if disease progression continued after four cycles of Sintilimab, with 200 mg Sintilimab used for maintenance therapy.

Ruxolitinib tablets (manufactured by Novartis Pharma Schweiz AG, specification: 5 mg per tablet) were dosed based on platelet count (PLT). For PLT < 50 × 10⁹/L, the dosage was 5 mg once daily; for PLT > 50–75 × 10⁹/L, the dosage was 5 mg twice daily; for PLT 75–100 × 10⁹/L, the dosage was 10 mg twice daily; and for normal PLT, the dosage was 15 mg twice daily. Treatment was administered for a total of 4 weeks, with weekly monitoring of cytokine levels and adjustments to the regimen based on the treatment response. Patients 1 to 3 received Ruxolitinib (RUX) treatment, and Patient 4 declined RUX treatment.

### Observational indicators

Basic information includes patient age, gender, and disease type. Clinical efficacy is defined as follows: Complete Remission (CR) is characterized by the disappearance of all clinical symptoms and signs, and normalization of sCD25, blood cell counts, and other abnormal laboratory parameters. Partial Remission (PR) is defined as a ≥ 25% improvement in two or more quantifiable symptoms and laboratory parameters. Ineffective treatment is when neither CR nor PR criteria are met.

Survival status includes median follow-up time, median survival time, overall survival rate (OS), median treatment duration, and median infusion dose. Dynamic changes in plasma EBV-DNA copy number were assessed by collecting 5 ml of fasting venous blood, centrifuging it, and storing the serum at -20 °C, with EBV-Sorting PCR used to detect target cells infected in the plasma.

Laboratory parameters such as serum ferritin (Ferr), alanine aminotransferase (ALT), aspartate transaminase (AST), triglycerides (TG), creatinine (Cr), interleukin-2 (IL-2), IL-4, IL-6, IL-10, interferon-γ (IFN-γ), tumor necrosis factor-α (TNF-α), fibrinogen (Fbg), and soluble CD25 (sCD25) were measured before and after PD-1 inhibitor treatment using enzyme-linked immunosorbent assay (ELISA) and the BK-1200 automated hematology analyzer. White blood cell count (WBC), hemoglobin (HGB), and platelet count (PLT) were also assessed, and peripheral blood lymphocyte subset changes, as well as B, T, and NK cell proportions, were analyzed using the Attune NxT flow cytometer.

Adverse reactions, including gastrointestinal issues and declines in liver and kidney function, were monitored, with treatment discontinued if grade ≥ 3 adverse reactions occurred, as detailed in Table 1.

**Table 1:** Classification of adverse reactions

Classification	Description
G1	Asymptomatic or mild symptoms; It can be self-alleviated without special intervention
G2	Mild limitations in daily activities; Need local treatment
G3	Disability or moderate-to-severe limitations in daily activities that are not life-threatening; Require hospitalization
G4	This is a life-threatening condition requiring immediate emergency treatment
G5	Death

### Statistical analysis

All data were processed using SPSS version 26.0. Categorical data were recorded as “n (%)” and analyzed using the chi-square test. Continuous data were recorded as mean ± standard deviation (SD) and analyzed using the t-test. A significance level of *P* < 0.05 was considered statistically significant for comparing data. Statistical data on changes in lymphocyte subpopulations were limited, so no statistical analysis was performed, and only trends in changes were described.

## Results

### Analysis of baseline characteristics and treatment conditions

Of the 4 patients, 3 were male and 1 was female, with a median age of 46 years (range: 29–65 years). All patients were refractory/relapsed EBV-HLH with no prior immunosuppressive treatment history and had different concurrent complications. Patient 1 received 13 treatment cycles and achieved complete remission (CR). Patient 2 underwent 6 treatment cycles and also reached CR. Patient 3 had 2 treatment cycles before treatment interruption and loss to follow-up. Patient 4 completed 14 treatment cycles but ultimately succumbed to the disease. All patients received Sintilimab, with a total dosage ranging from 400 mg to 2800 mg, and plasma EBV-DNA copy numbers decreased significantly after treatment in all patients. No adverse reactions were observed in all patients, and detailed information is provided in Table 2.

**Table 2:** Analysis of patient baseline characteristics

Item	Patient1	Patient2	Patient3	Patient4
Age	65	29	52	40
Sex	male	male	male	female
Recurrent/refractory	yes	yes	yes	yes
Course of treatment	13	6	2	14
Total dosage(mg)	2600	1200	400	2800
Plasma EBV-DNA(Copies/ml) - pre-treatment	4.1 × 10⁴	2.3 × 10⁵	1.8 × 10³	9.6 × 10⁷
Plasma EBV-DNA(Copies/ml) - First negative conversion	5.4 × 10	3.49 × 10²	24.31	2.5 × 10²
Number of treatments for the first positive-to-negative experience	3	2	1	1
Spleen size(mm) - pre-treatment	48	42	51	44
Spleen size(mm) - Last review	38	38	40	34
Adverse reactions	null	null	null	null
Clinical outcome	CR	CR	Lost to follow-up	Death
Complications	Chronic bronchitis、Double kidney stones、Caval cerebral infarction	Chronic viral hepatitis B、Fatty liver	Sjogren’s syndrome、Hypertension Level 1 Extremely High Risk Group、Right kidney stones	α thalassemia
Prior immunosuppressive treatments	No	No	No	No

### Analysis of follow-up and survival data

**Fig. 1:**
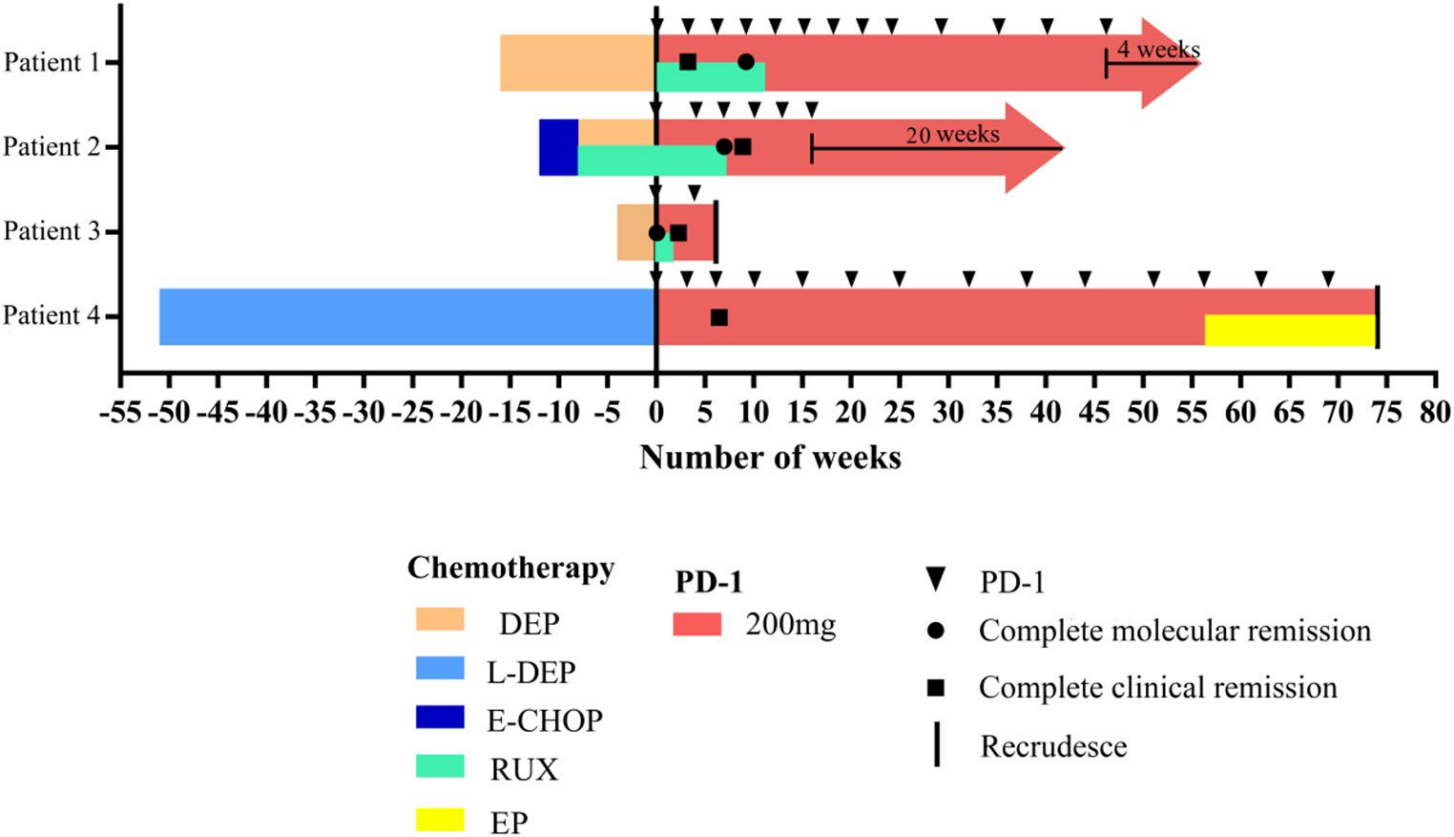
Patient Survival Status

Among the 4 patients, the median follow-up time was 43 weeks (range: 6–74 weeks). The median survival time was 14.25 months, with a 1-year overall survival (OS) rate of 75.00%. All 4 patients were treated with Sintilimab, with a median of 9.5 treatment cycles (range: 2–14) and a median infusion dose of 1900 mg (range: 400–2800 mg), as shown in Fig. 1.

### Dynamic changes in plasma EBV-DNA copy numbers

**Fig. 2:**
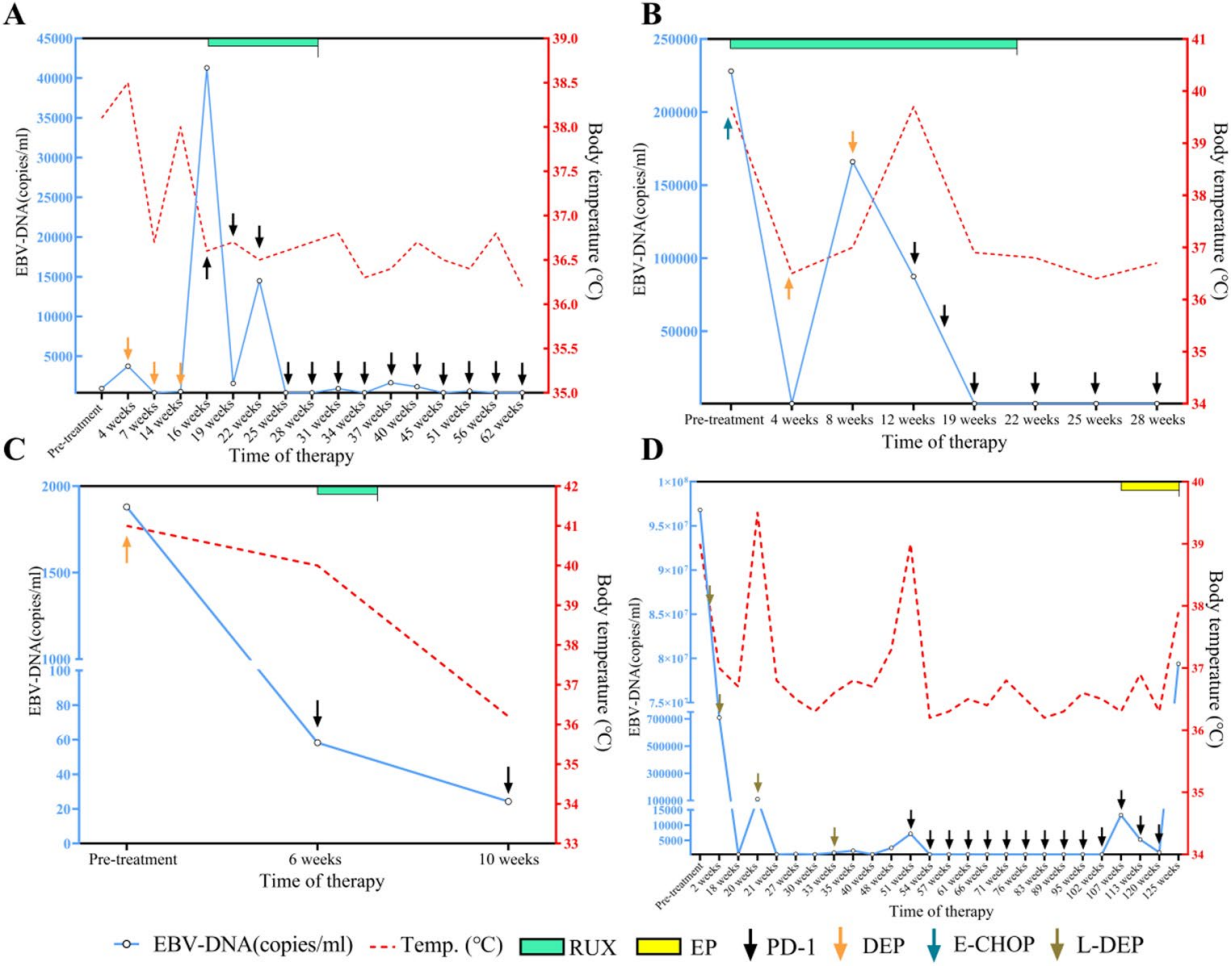
Dynamic changes in plasma EBV-DNA copy numbers in patients **A**: *Patient 1;* **B**: *Patient 2;* **C**: *Patient 3;* **D**: *Patient 4*

Dynamics of EBV-DNA Copy Number and Patient Outcomes shows the changes in EBV-DNA copy number over time for each patient, reflecting their treatment responses and disease progression. Patient 1’s EBV-DNA copy number peaked at 16 weeks of treatment, then rapidly declined but showed some fluctuations. After 37 weeks, it continued to decrease, and the patient achieved and maintained complete remission (CR) for 40 weeks following Sintilimab infusion, continuing to date. Patient 2’s EBV-DNA became undetectable after 4 weeks of treatment, with significant fluctuations in viral load at 8 weeks. The levels decreased afterward, and EBV-DNA became undetectable again at 19 weeks, resulting in CR. For Patient 3, the EBV-DNA copy number continuously decreased within the first 10 weeks of treatment, but the patient experienced treatment interruption due to non-adherence and was lost to follow-up. Patient 4’s EBV-DNA became undetectable at 18 weeks, but there were fluctuations afterward. The patient achieved undetectable EBV-DNA again at 54 weeks and maintained a stable condition until 102 weeks, when they died from multi-organ failure caused by a COVID-19-induced pulmonary infection(Fig. 2A-D).

### Dynamic changes in laboratory parameters

#### Blood cell parameters before and after PD-1 inhibitor treatment

There were no statistically significant differences in Ferritin (Ferr), Triglycerides (TG), Creatinine (Cr), and Fibrinogen (Fbg) between pre-treatment and after one cycle of PD-1 inhibitor therapy (*P* > 0.05). However, after one cycle of PD-1 inhibitor treatment, levels of Alanine Aminotransferase (ALT), Aspartate Aminotransferase (AST), and Interleukin-10 (IL-10) were significantly lower, while White Blood Cell count (WBC), Hemoglobin (HGB), and Platelet count (PLT) were significantly higher compared to pre-treatment levels (*P* < 0.05), as detailed in Table 3.

**Table 3:** Comparison of blood cell parameters before and after PD-1 inhibitor treatment (x ± s)

Index	*n*	Pre-treatment	1 course of treatment	t	*P*
Ferr(ng/ml)	4	12359.50 ± 10720.19	441.66 ± 543.11	2.221	0.068
ALT(U/L)	4	168.23 ± 61.13	16.70 ± 5.55	4.937	0.003
AST(U/L)	4	333.96 ± 211.47	27.98 ± 12.03	2.889	0.028
TG (mmol/L)	4	2.28 ± 0.69	1.78 ± 0.23	1.375	0.218
Cr(umol/L)	4	72.50 ± 12.29	70.75 ± 24.70	0.127	0.903
IL-10(pg/ml)	4	1017.98 ± 244.89	2.88 ± 1.15	8.290	< 0.001
Fbg (g/L)	4	1.63 ± 1.21	3.19 ± 1.81	1.433	0.202
WBC(×10⁹/L)	4	0.50 ± 0.32	4.30 ± 1.28	5.760	0.001
HGB(g/L)	4	58.00 ± 8.98	95.25 ± 14.64	4.338	0.005
PLT(×10⁹/L)	4	32.25 ± 15.44	210.50 ± 66.27	5.239	0.002

#### Complete blood cell count and sCD25

**Fig. 3:**
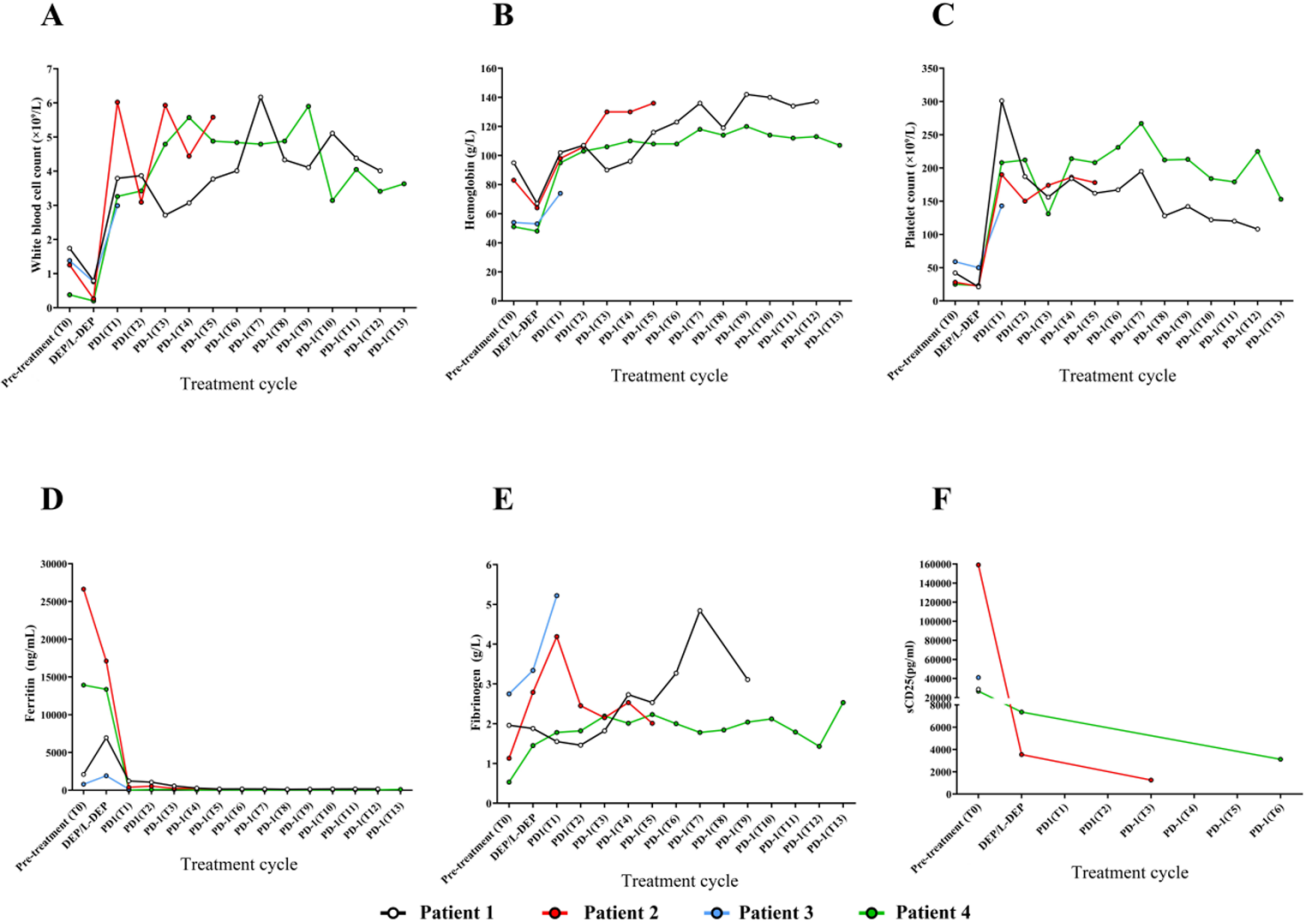
Dynamic Changes in Complete Blood Cell Count and sCD25 in Patients **A**: *WBC; ***B**: *HGB; ***C**: *PLT: ***D**: *Ferr; ***E**: *Fbg; ***F**: *sCD25*

After one cycle of PD-1 inhibitor treatment, there was a significant increase in WBC, HGB, and PLT for Patients 1 through 4, as shown in Figs. 3A-C. Ferritin (Ferr) levels decreased to the normal range, illustrated in Fig. 3D. Fibrinogen (Fbg) also showed an increasing trend, depicted in Fig. 3E. While sCD25 levels were not measured before treatment for Patients 2 and 4, Patient 2’s sCD25 levels rapidly declined after Ruxolitinib (RUX) combined with the DEP regimen. Patient 4 showed a less pronounced decrease in sCD25 levels following L-DEP chemotherapy, but sCD25 levels further declined with PD-1 inhibitor treatment. sCD25 levels were not available for Patients 1 and 3 during treatment, as shown in Fig. 3F.

#### Cytokine levels

After one cycle of Ruxolitinib (RUX) combined with PD-1 inhibitor treatment, levels of IL-2, IL-4, IL-6, IL-10, and IFN-γ decreased in Patients 1 and 2, while in Patient 3, IL-2, IL-6, IL-10, and IFN-γ also decreased. Additionally, TNF-α levels decreased in Patient 2. However, TNF-α levels increased in Patients 1 and 3 following the same treatment regimen. In Patient 4, IL-2 and IFN-γ levels increased after four cycles of the DEP regimen alone, while IL-4, IL-6, IL-10, and TNF-α levels decreased, as shown in Table 4.

**Table 4:** Changes of cytokine levels before and after treatment (pg/ml)

Index	Patient1	Patient1	Patient1	Patient1	Patient2	Patient2	Patient2	Patient2	Patient2	Patient3	Patient3	Patient4	Patient4
	pre-treatment	DEP(T2)	RUX PD-1(T1)	PD-1(T9)	pre-treatment	DEP RUX(T1)	DEP RUX(T2)	PD-1 RUX(T1)	PD-1(T3)	DEP(T1)	RUX PD-1(T1)	pre-treatment	DEP(T4)
IL-2	0.2	0.5	0.2	0.7	0.3	0.2	0.4	0.2	0.1	0.1	0.1	0.7	10.2
IL-4	0.5	0.6	0.4	0.4	0.2	0.3	0.4	0.3	0.3	0.3	0.4	7.9	3.8
IL-6	16.6	2.1	1.3	2.1	21.6	9.7	4.0	1.6	0.6	246.2	5.9	19.9	9.4
IL-10	1034.9	1232.0	1.7	2.3	1253.9	219.0	175.1	4.0	1.5	765.0	3.0	51.7	3.2
IFN-γ	382.4	151.3	34.0	76.0	6624.5	3314.0	1503.0	7.3	25.3	281.0	29.1	191.6	507.0
TNF-α	25.7	11.6	28.1	97.7	1.1	1.5	9.0	1.2	17.0	1.2	16.7	61.5	10.3
*T stands for times*													

#### Changes in peripheral blood lymphocyte subpopulations

**Fig. 4:**
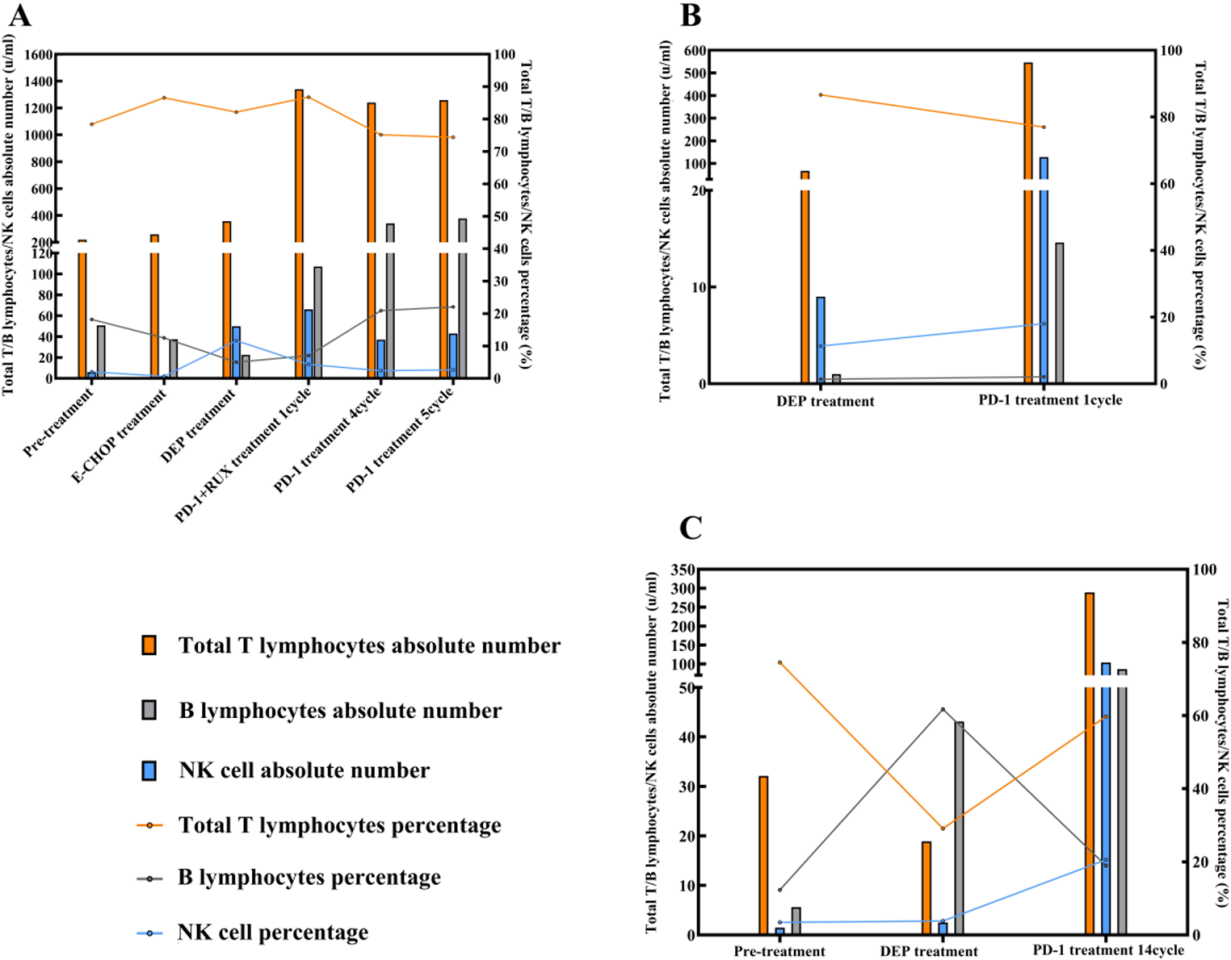
Changes in Peripheral Blood Lymphocyte Subpopulations in Patients **A**: *Patient 2; ***B**: *Patient 3*; **C**: *Patient 4*

Patient 1 declined the analysis of peripheral blood lymphocyte subsets. However, in Patients 2 through 4, following PD-1 inhibitor treatment, there was an increase in the number of T cells, B cells, and NK cells, with T cell expansion being the most pronounced. The proportion of T lymphocytes showed a decreasing trend, while the proportions of B cells and NK cells increased, as illustrated in Fig. 4A-C.

### Adverse reactions

**Fig. 5:**
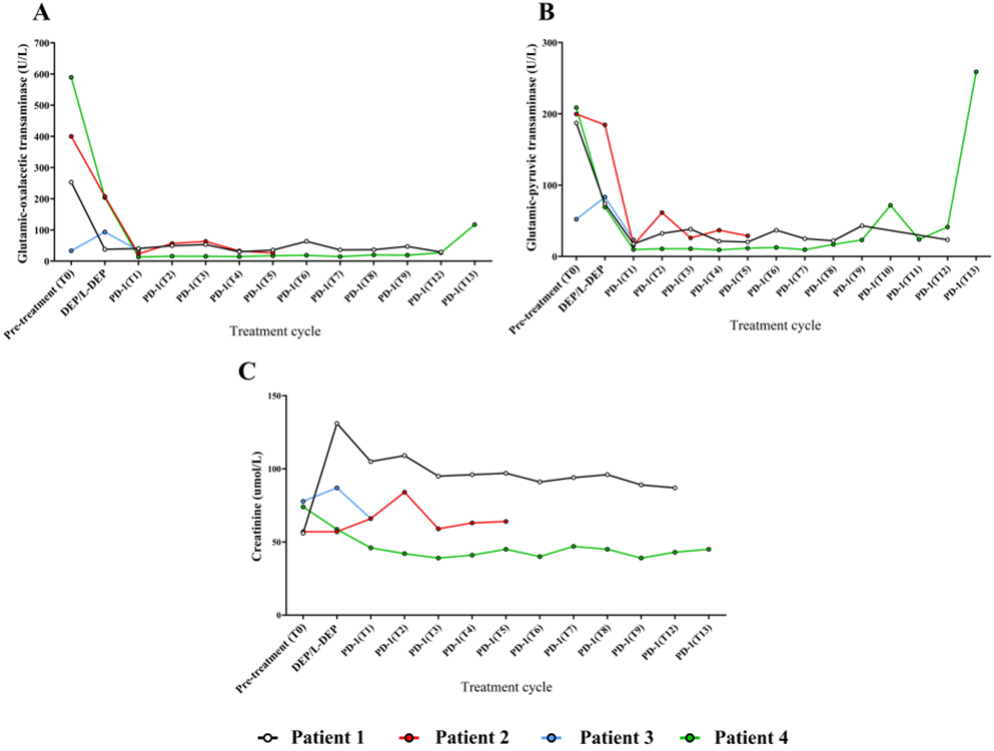
Changes in Liver and Renal Function During Treatment in EBV-HLH Patients **A**: *AST; ***B**: *ALT; ***C**: *Cr*

None of the patients experienced drug-related severe adverse reactions of grade 3 or higher. Liver and kidney function remained within normal ranges for Patients 1 through 3. Patient 4 experienced a temporary mild increase in ALT levels during maintenance therapy, as shown in Fig. 5.

## Discussion

### Current research status of EBV-HLH

A study by Shabbir M et al. [16] reported a mortality rate of 72% (13/18) in adults with hemophagocytic lymphohistiocytosis (HLH), with a median survival time of 35 days post-diagnosis, and multiple organ failure being the most common cause of death. Currently, there is no standard treatment for adult Epstein-Barr virus-associated HLH (EBV-HLH), and conventional chemotherapy, while targeting overactive immune cells, also weakens the patient’s immune system, exacerbating the EBV infection. Allogeneic hematopoietic stem cell transplantation is currently the only curative method for EBV-HLH [17]. However, the clinical application of allogeneic stem cell transplantation is limited by donor availability and can significantly impact the patient’s quality of life. Therefore, there is an urgent need for better strategies to treat EBV-HLH.

Simultaneously, after EBV infects CTLs, it can alter the antigenic determinants on the cell surface, enabling the corresponding receptors to bind with cytokines, leading to the activation of intracellular protein kinases, increasing cytokine secretion and release, and thereby exacerbating the cytokine storm through the activation of the JAK-STAT pathway [18]. RUX works by blocking the JAK1/2 pathway to reduce various cytokines and control the hyperinflammatory response, thereby alleviating HLH [19]. Research by Zhang Q et al. [20] has demonstrated that RUX is both safe and effective in treating pediatric HLH, with a CR rate of 73.1%, 42.3% of patients achieving sustained CR, and an ORR of 87.5% in EBV-HLH patients, with good tolerance. Moreover, PD-1 inhibitors can restore the killing activity of functionally exhausted CTLs, control viral load, and slow the clinical progression of HLH [21]. In 2022, Beijing Friendship Hospital reported a successful case of treating EBV-HLH with a PD-1 inhibitor [22]. This study found that a non-chemotherapy regimen combining RUX and sintilimab was effective in clearing EBV-HLH viral load, reducing pathological cytokines, and suppressing inflammation. Additionally, two patients achieved sustained clinical remission, and patients 1–3 showed good tolerance to the RUX and sintilimab regimen, with none of the four patients experiencing drug-related adverse reactions of grade 3 or higher. Thus, PD-1 inhibitors, either alone or combined with RUX, can normalize blood counts in a short time and promote the gradual recovery of cytokines and organ functions.

### Therapeutic value of PD-1 inhibitors combined with RUX in treating EBV-HLH

A study involving 61 cases of EBV-HLH reported that the one-year OS for EBV-HLH patients was only 25% [23]. In this study, the one-year OS was 75% after using PD-1 inhibitors alone or in combination with RUX. Specifically, the efficacy for cases 1 and 2 was maintained until the 50th and 36th weeks after the initial treatment, respectively, without relapse. Case 3 showed a significant decrease in EBV-DNA copies during the two treatment cycles but discontinued treatment afterward due to non-adherence. Case 4 ultimately died due to COVID-19-induced pneumonia and multiple organ failure, which was related to chemotherapy suppressing the patient’s immune function.

However, after one treatment cycle with PD-1, ALT, AST, and IL-10 levels were significantly lower than before PD-1 inhibitor treatment, while WBC, HGB, and PLT were significantly higher. This suggests that PD-1 inhibitors, either alone or in combination with RUX, control the inflammatory storm faster than traditional chemotherapy regimens, with less bone marrow toxicity and a lower risk of relapse. In this study, cases 2 and 4 both used the DEP regimen in the early stages, with the difference that case 2 also used RUX, and the decrease in sCD25 levels after treatment with the RUX combined DEP regimen was significantly better than in case 4, who declined RUX treatment. Additionally, case 2 showed a further downward trend in IL-2, IL-4, IL-6, IL-10, IFN-γ, and TNF-α levels after using PD-1 inhibitors, whereas case 4’s IL-2 and IFN-γ levels increased after four cycles of the DEP regimen alone. This indicates that the RUX combined with PD-1 inhibitors can significantly suppress the cytokine storm and improve the inflammatory activity of EBV-HLH.

Plasma EBV-DNA copy levels are a crucial indicator for evaluating the progression and efficacy of EBV-HLH treatment [24]. In the four cases studied, a significant decline in plasma EBV-DNA copies was observed after 1–2 treatment cycles with PD-1 inhibitors. Previous research has found that humanized mice infected with EBV exhibited high mortality and characteristics of EBV-HLH, while heat-inactivated EBV did not trigger any noticeable symptoms. This suggests that the development of HLH-like syndrome depends on excessive viral replication [25]. This indicates that early use of PD-1 inhibitors to control viral load may alleviate the progression of HLH. However, in case 4, plasma EBV-DNA reappeared later in treatment, likely due to the high prevalence of EBV infections in the population, making recurrent infections difficult to avoid. Additionally, after contracting COVID-19, the patient’s organ function may have further declined, leading to a poor prognosis.

In peripheral blood lymphocyte analysis, except for case 1 who declined testing, cases 2 and 3, treated with PD-1 inhibitors combined with RUX, and case 4, treated with PD-1 inhibitors alone, all showed an increase in T cells, B cells, and NK cells after treatment, with a rising trend in the proportion of B cells and NK cells. This suggests that PD-1 inhibitors, either alone or in combination with RUX, can directly improve immune function, and lymphocyte proliferation is likely a key mechanism for enhancing the body’s ability to clear EBV.

### Safety of PD-1 inhibitors combined with RUX in treating EBV-HLH

Under the continuous stimulation from infections, tumors, and other factors, T cells undergo persistent proliferation and excessive Th1 activation, leading to an imbalance between Th1 and Th2 cells. This results in the production of large amounts of IFN-α, activation of CD8 + T cells, and the generation of various interleukins and other chemotactic factors that infiltrate various organ tissues, causing systemic symptoms [26]. These symptoms can include fever, pancytopenia, hypertriglyceridemia, hypofibrinogenemia, hepatosplenomegaly, liver function abnormalities, and even disseminated intravascular coagulation (DIC) and multi-organ failure (MOF) [27]. Additionally, HLH can be categorized into various types based on the defective genes, such as familial hemophagocytic lymphohistiocytosis type 3 (FHL-3), which leads to coagulopathy and impaired intracellular bacterial killing; familial hemophagocytic lymphohistiocytosis type 4 (FHL-4), which reduces the cytotoxic function of CTL and NK cells; and familial hemophagocytic lymphohistiocytosis type 5 (FHL-5), which can induce anemia due to abnormalities in erythrocyte production, maturation, and morphology [28–30]. Immune deficiency-related HLH can also lead to progressive neurological disorders, impaired platelet aggregation, and neutropenia [31–33].

The use of chemotherapy drugs often exacerbates or induces additional cellular dysfunction and toxic side effects in the short term. However, throughout the treatment process, no severe life-threatening drug-related adverse events were observed in the four cases, with only case 4 experiencing a transient mild increase in ALT during maintenance therapy, indicating that the combination of PD-1 inhibitors and RUX has significant therapeutic potential for EBV-HLH with a good safety profile.

## Conclusions

Treatment of adult EBV-HLH with PD-1 inhibitors alone or in combination with RUX promotes rapid blood cell recovery as well as expansion of T, B, and NK lymphocytes. It effectively reduces plasma EBV-DNA copy levels, controls a range of symptoms caused by cytokine storms in the short term, and demonstrates good tolerability and safety. This approach not only improves remission rates for newly diagnosed and relapsed/refractory EBV-HLH, extending patient survival and progression-free survival, but also offers an alternative treatment option for patients who are ineligible for or refuse transplantation.

However, EBV-HLH is a rare and highly fatal condition, and this study includes a small sample size, necessitating further research with a larger cohort to validate its efficacy and safety. Additionally, the mechanisms of the PD-1 inhibitor and RUX combination therapy have not been fully explored, and the study did not assess the expanded cell subtypes. Further investigation is needed to determine whether this new regimen could potentially become a non-chemotherapy curative option for EBV-HLH, whether PD-1 inhibitors can be discontinued after controlling hemophagocytic activity, how long maintenance therapy should continue, the criteria for discontinuation, and the long-term efficacy for patients.

## Data Availability

The datasets used or analysed during the current study are available from the corresponding author on reasonable request.
